# The role of self-reported fear and disgust in the activation of behavioral harm avoidance related to medical settings

**DOI:** 10.3389/fpsyt.2023.1074370

**Published:** 2023-01-24

**Authors:** Béla Birkás, Botond Kiss, Carlos M. Coelho, András N. Zsidó

**Affiliations:** ^1^Department of Behavioural Sciences, Medical School, University of Pécs, Pécs, Hungary; ^2^Faculty of Human and Social Sciences, Institute of Psychology, University of Pécs, Pécs, Hungary; ^3^Department of Psychology, Faculty of Human and Social Sciences, Azores University, Ponta Delgada, Portugal; ^4^Center for Psychology, Porto University, Porto, Portugal; ^5^Szentagothai Research Centre, University of Pécs, Pécs, Hungary

**Keywords:** blood-injection-injury phobia, perceived control, fear of pain, life history strategies, experience, knowledge

## Abstract

**Introduction:**

Although adaptive defense mechanisms are useful in helping us avoid getting injured, they are also triggered by medical interventions and procedures, when avoidance is harmful. A body of previous results showed that both fear and disgust play a pivotal role in medical avoidance. However, the underlying mechanisms are not fully understood. Thus, the aim of the current study was to examine the effects of experience, perceived control, and pain on medical avoidance with disgust and fear as mediating factors from an evolutionary perspective.

**Methods:**

We assessed participants' knowledge of and experience with medical procedures, former negative medical experiences, and health-related information; their life history strategy variation; pain-related fear and anxiety of medical procedures; perceived control over emotional reactions and extreme threats; disgust sensitivity; blood-injury-injection phobia and medical treatment avoidance.

**Results:**

We found that more knowledge, experience, and a slower life strategy were linked to a greater level of perceived control and attenuated emotional reactions. Further, better ability to control affective and stress reactions to negative experiences was linked to reduced disgust and fear of pain, and thus might mitigate the level of perceived threat, and diminish fear and disgust reactions.

**Discussion:**

More knowledge and experiences, better perceived control together with reduced disgust and fear of pain can decrease the probability of avoiding medical situations. Implications to treatment are discussed. Results support the importance of targeting these contextual factors in prevention to increase the likelihood of people attending regular screenings or seeking medical care when needed.

## Introduction

Adaptive defense mechanisms facilitate recognition and appropriate responses to potential environmental threats that may cause injuries or even death [e.g., (1–3)]. Evolved defensive strategies include learned avoidance behaviors that are aimed to reduce the probability of close encounters with the individual's natural enemies (4), pathogens (5), and dangerous situations in general (6). Relevant research has a basic premise that adaptations and selection mechanisms build physiological and behavioral traits to enhance the adjustment of the organism to the environment in which they live. Life History Theory (LHT) (7) provides a framework to describe different fitness maximizing strategies and their adjustment to local conditions (8). Individual Life History (LH) strategies are shaped mainly by the controllability and predictability of life events (7, 9, 10). More uncontrollable and/or unpredictable circumstances are aversive and increase mortality and morbidity by factors such as pathogen prevalence, resource scarcity and other environmental threats (11). Thus, more unpredictable conditions (which are also more uncontrollable) are strongly associated with elevated environmental risks for the organism (e.g., harshness) and have detrimental effects on both, physical and mental health (12). Psychological health is also affected by the relatively low impact of the organism's behavior on the likelihood of these unpredictable and uncontrollable events, because it limits the development of adaptive behavioral strategies to avoid or escape subsequent similar situations in the future (9, 11, 12). Still, in less predictable and controllable conditions, avoidant strategies are beneficial, because of keeping the organism away from the threat, but these behaviors are more influenced by and vary across situations and conditions. Avoidance strategies for pathogens and injuries include mechanisms associated with fear and anxiety (e.g., predation or injury risk), and disgust (4, 13, 14). Conceptualizing fear as a defensive response to eminent threats and anxiety as a response to potential threats (15), these notions share similarities with disgust propensity. As suggested by the *disease-avoidance model* (16), disgust is an adaptive response to sources of potential contaminants and pathogens, protecting from infectious diseases (17, 18). Both direct encounters with natural enemies and indirect cues associated with various sources of risks (e.g., without the actual presence of enemies) can activate physiological mechanisms and behavioral strategies of avoidance leading to certain fitness costs to the individual through physiological (e.g., stress) and behavioral (e.g., aversion of certain places) effects [see (19)]. The neurobiological basis of approach and avoidance behavior, suggested by the revised Reinforcement Sensitivity Theory (20, 21) consists of three overlapping, but different affective-motivational systems. The *Fight-Flight-Freeze System* (FFFS) is the primary system responsible for active avoidance and escape behaviors in response to fear, thus, it is sensitive to aversive stimuli, both unconditioned (innate) or conditioned (learned). The activation of the FFFS is associated with a desire to escape and an emotional state corresponding primarily to anxiety and fear. At the behavioral level, the FFFS is expressed in active avoidance and escape. Together, these are related to panic and phobia on a psychopathological level (22). Disgust appears to be involved in this system since individuals with higher levels of anxiety show increased disgust sensitivity and avoidant behaviors (23, 24). Moreover, disgust is suggested to be associated not only with contamination-related or other specific avoidance but also with more general forms of behavioral inhibition, similar to fear (24, 25). To minimize the risk of exposure to pathogens and the chance of contamination, it is adaptive to sensitize disgust reactions toward the mere potential of infection and trigger avoidance in more general situations, rather than only if detecting actual pathogens (26). More recent findings support this notion showing, that disgust elicits more cognitive inhibition (i.e., attentional avoidance), whereas fear is associated with more cognitive focus on the triggering stimuli [see (27)], but both lead to similar reactions on the behavioral level [e.g., behavioral inhibition; see (25)]. Nonetheless, disgust-related responses are more specific and context-dependent, than the fear-related components of avoidant behavior (28).

Medical interventions and procedures carry the possibility of violating the integrity of the body and increase the likelihood of infections; similarly, to getting injured (29). Medical fears, that is fear of blood, injury and injections (BII) can also affect individuals' motivation to either seek or procrastinate medical help and care (30, 31). These medical fears are associated with excessive distress toward stimuli commonly present in medical settings [e.g.: vaccination, symptoms of illness, medical devices, see: (32, 33)] as well as disgust-related responses [see (34)]. Distress and disgust associated with medical situations often result in avoidance, even when medical treatment is needed (35). Being one of the most common specific phobias, BII-related fears have the most serious potential consequences to health affecting a large number of people (30, 36). The evolved avoidance mechanisms to deal with potential contamination can also be triggered when they should not. The disease-avoidance model, indeed, describes the functional role of disgust in the avoidance of contamination and protection of the individual against infections (16, 37). Accordingly, a large body of studies demonstrated that disgust is a critical factor in medical fear-related disorders [see (38) for a review] and consequently, underscored the relevance of this reaction in understanding medical avoidance. Medical care avoidance not only increases morbidity and mortality risk associated with treatable and preventable health conditions (39, 40), but it also intensifies negative psychological outcomes such as anxiety and depression (41, 42), and elevates healthcare-related economic costs. Treatment avoidance is suggested to be affected by both external (e.g., socioeconomic or demographic) and internal (i.e., knowledge, experiences, pain sensitivity) factors (43–46).

One of the most straightforward signals of medical need and, at the same time, one of the most powerful aversive stimuli is pain. Consequently, humans are characterized by vigilance to pain, that expedites escape and avoidance behavior (47) and is closely related to fear (48). Pain is crucial for survival due to its signal function alerting the individual to potential damages and activating behavioral responses to prevent or limit subsequent harms or injuries (49). Perception of pain is modulated by a wide range of environmental and psychological factors including previous experience, socioeconomic factors, education, health literacy, stressful life events, anxiety, and fear (50). Uncontrollability and helplessness are also dominant elements of fears and phobias and are strongly linked to avoidance (51). Accordingly, the personal appraisal of pain and its potential consequences affect not only the perception of pain but the associated avoidant behavioral responses as well. Perceived lack of control related to painful experiences elevate the negative orientation toward pain [i.e., pain catastrophizing; see (46)]. This intensifies negative expectations regarding subsequent pain-related experiences and results in evaluating potential consequences of pain as more threatening and potentially harmful. Anticipation of potential threats increases the level of anxiety and may even induce disgust, and, hence, facilitates the development of pain-related fear (52, 53). To sum up, the way individuals interpret pain, and its potential consequences, affects fear of pain (Algophobia) and related defense behaviors, such as avoidance enabling the person to memorize and later avoid cues associated with pain.

Health-related knowledge and former medical experience may alter the effects of helplessness and uncontrollability in both negative and positive ways. *Fear-avoidance models* suggest that fear of pain is an important component of pain chronification and related disabilities (52). Individuals suffering from pain commonly experience feelings of helplessness and uncontrollability, eliciting emotions of embarrassment or shame fuelled by individual and interpersonal worries (e.g., being unable to work; being a burden for the family) (54). Uncontrollability and helplessness are also key determinants of cognitive pain processing and have detrimental physical and psychological health consequences (55). Negative experiences or lack of understanding of symptoms enhances the level of perceived lack of control and vulnerability. However, a large body of previous evidence shows that positive outcomes of former medical care and a higher level of health literacy are associated with an increased sense of control and resilience (56, 57). Health-related knowledge and medical experiences were also found to affect the severity of chronic illnesses, procrastination of healthcare utilization, and biopsychosocial consequences of symptoms (43–45).

Hence, the overarching goal of the current study was (1) to examine the effects of medical fear and fear of pain on medical avoidance with (2) respect to disgust sensitivity, whilst controlling for former experiences, level of medical knowledge, perceived control, and life-history strategies. Former studies overlooked the evolutionary aspects of medical avoidance or did not include both, fear and disgust in the examination, or lack of information related to participants' medical knowledge and/or experiences. To prevent the severe consequences of medical avoidance, the identification of relevant risk factors of medical avoidance and exploring the interplay between disgust and fear is important. More specifically, based on the literature reviewed, we hypothesized that:

1. Former negative medical experiences, lack of health-related knowledge, exaggerate medical fears, and fear of pain.
2. Fear of pain will trigger individuals to perceive medical situations as more threatening and potentially harmful, which, in turn, will activate avoidance.
3. Disgust will be enhanced by indicators of uncontrollability and increases perceived threat, which, in turn, leads to elevated medical fears and medical avoidance.

## Methods

### Participants

We recruited 906 Caucasian participants (233 males, 662 females, 11 preferred not to answer), aged 18-68 years (M = 24.83, SD = 7.87) through the Internet by posting invitations on various forums and mailing lists to obtain a heterogeneous sample. The required sample size for this experiment was determined by computing estimated statistical power with a conservative approach (RMSEA = 0.035, 1-β=0.95, df = 103) using the semPower package for R (58, 59). The analysis indicated a required minimum total sample size of 458; thus, our study was adequately powered. The data were collected from January to March 2021. Regarding COVID-19 regulations, there was no curfew at the time of the data collection and medical examinations were freely available for everyone. The participants filled out the questionnaires online in Hungarian (their native language), using Google Forms, on a voluntary basis. Where the Hungarian version was not available, we translated the original English versions following the APA guidelines for translating psychological assessments. None of them reported having a psychiatric disorder. Additionally, we deleted three invalid entries (two duplications and one fake), thus they were not analyzed and are not included in the number of participants or mean age. The research was approved by the Hungarian United Ethical Review Committee for Research in Psychology and was carried out bythe Code of Ethics of the World Medical Association (Declaration of Helsinki). Informed consent was obtained from all participants.

### Materials

#### Demographic information

Demographic questions included age, gender, and inquiries about experience and knowledge of medical procedures, examinations, and injuries. We assessed participants' *knowledge* of medical procedures, examinations, and injuries with questions about their healthcare-related studies (e.g., first aid, nursing, medical doctor) and jobs in a healthcare-related field. Participant's *experience* with medical procedures, examinations, and injuries was assessed with questions about how often they go to examinations, screenings, blood draws, blood plasma donation, receive injections; how often they see serious injuries or blood in person or in the media (e.g., movies, Internet); whether they ever had a serious injury; and whether they had to provide care for a relative for at least one month. We also collected information regarding the quality of the last medical encounter of the participant as a patient. Questions regarding how satisfied (on a 1-5 scale) the person was with the level of respect and care of the visit and whether or not he/she would like to have the same level of respect and care for the next visit were asked but also excluded from the analysis. The results linked to these questions were weak or negligible (for satisfaction: *rs* = −0.132 to 0.003; for next visit expectations: Cohen's *d*s = −0.019 to 0.018), providing no additional information to our model, thus we decided not to include these into the statistical analysis.

#### Life history strategy variation

To indicate the adaptive strategies of the individual shaped by avoidance-related experiences and the levels of unpredictability and uncontrollability during childhood, we used the Mini-K scale of the Arizona Life History Battery (60), a brief 20-item measuring fast–slow life history strategies. Participants responded on 7-point Likert-type scales, mean scores were calculated across all items where higher scores indicated a slower life strategy. In this study, McDonald's omega was 0.75.

#### Fear of pain

To assess pain-related fear and anxiety of medical procedures, we used the Fear of Medical/Dental Pain (FPQ MD) scale of the brief Fear of Pain Questionnaire (61). Participants responded on 5-point Likert-type scales, sum scores were calculated across all items, and higher scores indicated higher levels of fear. In this study, McDonald's omega was 0.76.

#### Perceived control

We used the Anxiety Control Questionnaire (ACQ) to measure perceived control over emotional reactions and extreme threats (62). The ACQ is a 15-item questionnaire that has three subscales: emotion control, threat control, and stress control. Participants responded on 6-point Likert-type scales, sum scores were calculated for each subscale, and higher scores indicated higher levels of perceived control. In this study, McDonald's omegas were 0.81 (emotion), 0.78 (threat), and 0.74 (stress).

#### Disgust sensitivity

To assess disgust sensitivity, we used the revised version of the Disgust Scale-Revised (DSR) (63). The DSR has 25 items and taps into three dimensions of disgust: core disgust, animal reminder disgust (AR), and contamination-based disgust. From the 25 questions, 13 are true–false items (scored 0 or 1), and 12 items are rated on a 3-point Likert-type scales scale referring to the extent to which participants find a given experience disgusting (i.e., not, slightly, very; scored 0, 0.5, 1, respectively). Mean scores were calculated for each subscale, higher scores indicated higher levels of disgust sensitivity. In this study, McDonald's omegas were 0.63 (core), 0.60 (animal reminder), and 0.61 (contamination-based).

#### Medical fears

The short version of the Medical Fear Survey (MFS-short) is a 25-item questionnaire measuring medically related fears across five domains: injections and blood draws (IBD), sharp objects (SO), blood (BL), mutilation (MU), and examinations and symptoms (ES) (64). Participants rated the items using 4-point Likert-type scales. We calculated sum scores for each subscale, higher scores indicated higher levels of fear. In this study, McDonald's omegas were 0.88 (injections and blood draws), 0.83 (sharp objects), 0.87 (blood), 0.82 (mutilation), and 0.79 (examinations and symptoms).

#### Avoidance

We measured the extent to which participants have avoided medical treatment due to fear of various procedures and anticipated outcomes with two subscales (fear of finding a serious illness and fear of injections and pain) of the Medical Avoidance Survey (MAS) (65). The MAS has 21 items, rated on 5-point Likert-type scales. We calculated sum scores for each subscale, higher scores indicated higher levels of avoidance. In this study, McDonald's omegas were 0.85 (serious illness) and 0.84 (injections and pain).

#### Data analyses

First, we computed knowledge and experience scores separately but with the same method. We calculated the z scores for each variable and then added them up. The z transformation was necessary because not all questions were rated on the same length scale.

We performed a Structural Equation Modeling using the JASP statistical software version 0.15 for Windows (66) utilizing the lavaan (v. 0.6-1) package for R (67) to assess fit measures for our proposed models. Questionnaires with multiple scales (ACQ, DS, MFS, and MAS) were entered as latent variables. We used the diagonally weighted least squares (DWLS) estimator (68) with standard error calculation, and standard model test; missing data handling was listwise deletion (zero cases were removed). To evaluate model fit, we used the relative chi-square (?^2^/df), the comparative fit index (CFI), the Tucker–Lewis index (TLI), the root mean square error of approximation (RMSEA) with 90% confidence intervals, and the standardized root mean square residual (SRMR). The cut-offs for good model fit were a relative chi-square < 5 (36), CFI and TLI values of 0.95 or greater (69), RMSEA and SRMR values of 0.08 or lower (70).

## Results

**Figure 1:**
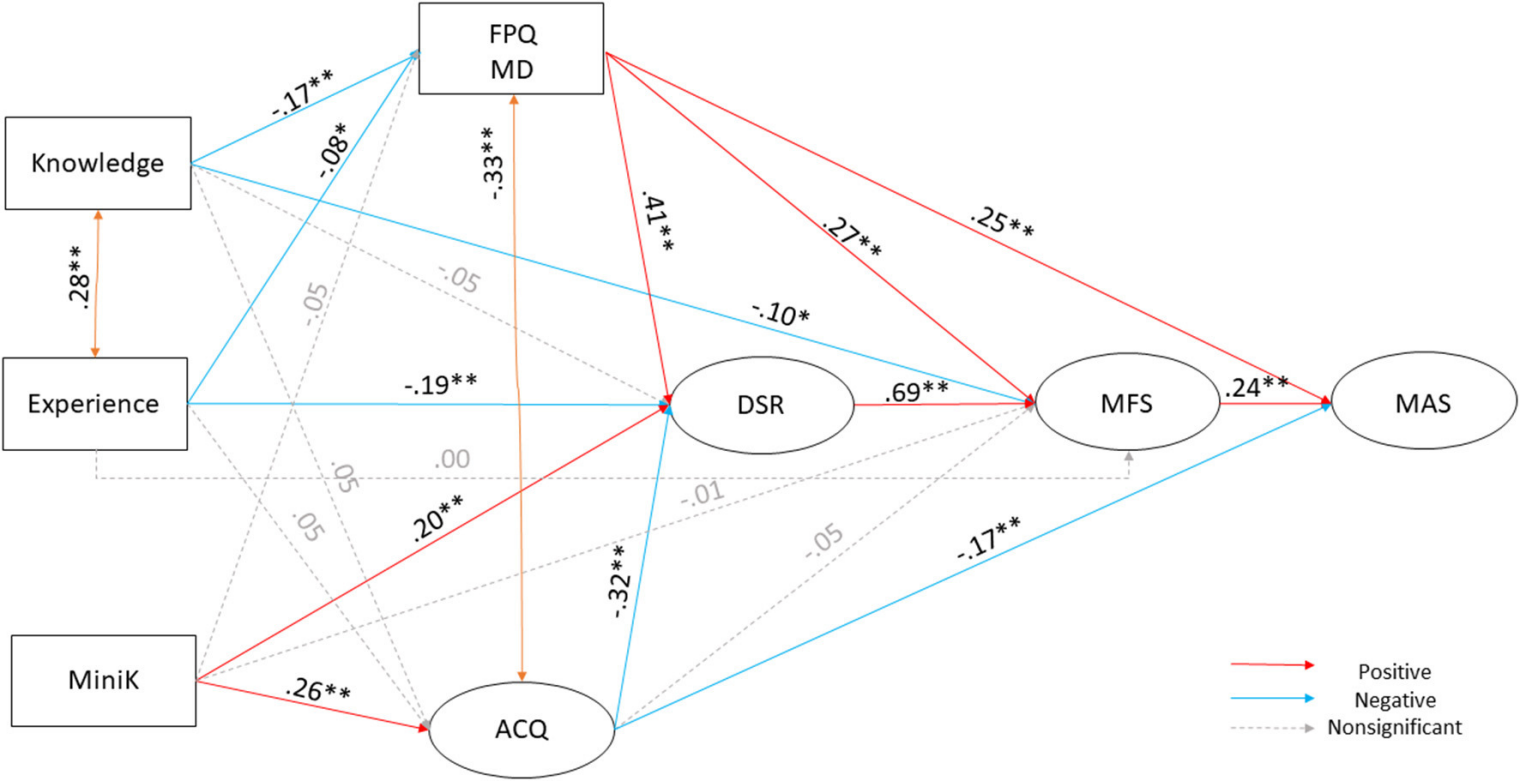
Our proposed model on the connection and background mechanisms of avoidance of health care due to fear and anxiety. Squares indicate measured variables; ellipses indicate latent variables (comprised of the subscale of the questionnaire). All pathways implemented in the Structural Equation modeling are presented in the figure. All reported estimates are the maximum likelihood standardized point estimates. Statistically significant unstandardized point estimates are indicated with a star (^*^p < 0.05, ^**^p < 0.001). Significant positive predictors are indicated with red color, significant negative predictors are indicated with blue color. Nonsignificant routes are indicated by dashed lines and gray color.

We sought to examine the effects of medical fear and fear of pain on medical avoidance with disgust sensitivity as mediating factor and also controlling for former experiences, level of medical knowledge, perceived control, and life-history strategies. We tested the hypothetical model shown in Figure 1. The test yielded a good model fit [^2^(100) = 343.28, *p* < 0.001, CFI = 0.97, TLI = 0.95, RMSEA = 0.05, 90% CI = [0.046–0.058], SRMR = 0.06). Fear of medical pain (FPQ MD) was negatively predicted by knowledge (β = −0.17, *p* < 0.001) and experience (β = −0.08, *p* = 0.032), but not by life history strategy variation (MiniK; β = −0.05, *p* = 0.13). Perceived control (ACQ) was positively predicted by Mini-K (β = 0.26, *p* < 0.001), the routes from knowledge (β = 0.05, *p* = 0.12) and experience (β = 0.05, *p* = 0.12) were non-significant. Disgust sensitivity (DSR) was positively predicted by fear of medical pain (β = 0.41, *p* < 0.001), and life history strategy variation (β = 0.20, *p* < 0.001), while experience (β = −0.19, *p* < 0.001) and perceived control (β = −0.32, *p* < 0.001) were negative predictors. Knowledge (β = −0.05, *p* = 0.26) did not have a significant effect on DSR. Medical fear (MFS) was positively predicted by fear of medical pain (β = 0.27, *p* < 0.001) and disgust sensitivity (β = 0.69, *p* < 0.001), while knowledge (β =−0.10, *p* = 0.010) predicted it negatively. The paths from experience (β = 0.004, *p* = 0.93), life history strategy variation (β =−0.01, *p* = 0.83) and perceived control (β = −0.05, *p* = 0.31) were non-significant. Medical avoidance (MAS; R^2^ = 0.29) was predicted positively by medical fear (β = 0.24, *p* < 0.001) and fear of medical pain (β = 0.25, *p* < 0.001), while perceived control (β = −0.17, *p* < 0.001) was a negative predictor.

We allowed covariances between some variables because they were on the same level of the model, and we found high correlations between them. Perceived control and fear of medical pain (β = −0.33, *p* < 0.001), and knowledge and experience (β = 0.28, *p* < 0.001) scores showed strong covariances as expected. We also controlled for covariance between two of the disgust sensitivity subscales animal remainder and contamination (β = 0.19, *p* < 0.001). Table 1 shows the descriptive statistics of the sample on all measures used; Table 2 shows the unstandardized coefficients and confidence intervals. See Table 3 for correlational coefficients between the variables included in the model.

**Table 1:** Descriptive statistics (means and standard deviations – SD) of the sample on disgust sensitivity (DS) core, animal remainder (AR), contamination; medical fear survey (MFS) injection and blood draw (IDB), sharp objects (SO), examinations and symptoms (ES), blood (BL), mutilation (MU); fear of medical/dental pain (FPQ MD); life history strategy variation (Mini-K); perceived control (ACQ) emotion (EC), threat (TC), and stress (SC); medical avoidance (MAS); age; number of respondents (separately for genders).

	**Mean**	**SD**
Experience	4.149	1.122
Knowledge	1.649	1.078
MiniK	5.086	0.697
FPQ_MD	7.872	3.103
ACQ_EC	12.486	5.514
ACQ_TC	18.533	5.348
ACQ_SC	7.369	3.308
DSR_core	0.501	0.149
DSR_AR	0.460	0.221
DSR_contam	0.395	0.199
MFS_IBD	2.550	3.142
MFS_SO	1.480	2.301
MFS_ES	7.301	3.497
MFS_BL	2.099	2.955
MFS_MU	7.286	4.065
MAS_SI	5.397	5.967
MAS_BI	2.930	3.870
Age	24.83	7.87
Gender	233 662 11	male female did not answer

**Table 2:** Detailed statistical results for the pathways in structural equation modeling with unstandardised point estimates (B), standard errors (SE), *z* and *p*-values, 95% confidence intervals (CI) for B values, and standardized estimates (β).

**Predictor**	**Outcome**	***B***	**SE**	***z*-value**	***p***	**95% Cl**		**β**
						**Lower**	**Upper**	
MFS	MAS	0.467	0.118	3.969	< 0.001	0.125	0.368	0.239
ACQ	MAS	−0.181	0.038	−4.738	< 0.001	−0.237	−0.098	−0.166
FPQ_MD	MAS	0.315	0.092	3.412	< 0.001	0.07	0.257	0.246
ACQ	DSR	−0.075	0.011	−6.987	< 0.001	−0.357	−0.201	−0.318
FPQ_MD	DSR	0.113	0.013	8.354	< 0.001	0.179	0.289	0.405
Knowledge	DSR	−0.039	0.034	−1.131	0.258	−0.076	0.021	−0.049
Experience	DSR	−0.146	0.034	−4.281	< 0.001	−0.16	−0.059	−0.19
MiniK	DSR	0.248	0.052	4.801	< 0.001	0.098	0.234	0.201
Knowledge	FPQ_MD	−0.5	0.106	−4.696	< 0.001	−0.246	−0.101	−0.174
Experience	FPQ_MD	−0.223	0.104	−2.145	0.032	−0.154	−0.007	−0.081
MiniK	FPQ_MD	−0.222	0.146	−1.515	0.13	−0.164	0.021	−0.05
DSR	MFS	1.621	0.233	6.944	< 0.001	0.552	0.986	0.69
ACQ	MFS	−0.029	0.029	−1.015	0.31	−0.15	0.048	−0.052
FPQ_MD	MFS	0.175	0.04	4.319	< 0.001	0.094	0.251	0.267
Knowledge	MFS	−0.188	0.073	−2.563	0.01	−0.114	−0.015	−0.1
Experience	MFS	0.007	0.082	0.084	0.933	−0.055	0.06	0.004
MiniK	MFS	−0.025	0.131	−0.191	0.849	−0.09	0.074	−0.009
Knowledge	ACQ	0.163	0.105	1.561	0.119	−0.008	0.072	0.049
Experience	ACQ	0.157	0.1	1.576	0.115	−0.008	0.072	0.049
MiniK	ACQ	1.362	0.147	9.249	< 0.001	0.195	0.299	0.262
***Covariance***								
Knowledge	Experience	0.338	0.047	7.193	< 0.001	0.203	0.355	0.279
ACQ	FPQ_MD	−3.442	0.359	−9.575	< 0.001	−4.146	−2.737	−0.328
DSR_core	DSR_contam	0.425	0.111	3.814	< 0.001	0.7	0.216	0.189

DS, Disgust sensitivity; AR, animal remainder contamination; MFS, Medical Fear Survey; FPQ MD, Fear of Medical/Dental Pain; MiniK, Life history strategy variation; ACQ, Perceived Control; MAS, Medical Avoidance.

**Table 3:** Pearson correlational coefficients between all variables used in the structural equation modeling.

**Variable**	**DSR** **core**	**DSR** **AR**	**DSR** **contam**	**MFS** **IBD**	**MFS** **SO**	**MFS** **ES**	**MFS** **BL**	**MFS** **MU**	**FPQ** **MedDent**	**MiniK**	**ACQ** **EC**	**ACQ** **TC**	**ACQ** **SC**	**MAS** **SI**	**MAS** **BI**	**Knowledge**
1. DSR_core	—															
2. DSR_AR	0.416^***^	—														
3. DSR_ contamination	0.363^***^	0.289^***^	—													
4. MFS_IBD	0.253^***^	0.333^***^	0.167^***^	—												
5. MFS_SO	0.318^***^	0.289^***^	0.212^***^	0.261^***^	—											
6. MFS_ES	0.310^***^	0.337^***^	0.230^***^	0.281^***^	0.290^***^	—										
7. MFS_BL	0.297^***^	0.433^***^	0.249^***^	0.558^***^	0.418^***^	0.349^***^	—									
8. MFS_MU	0.388^***^	0.584^***^	0.254^***^	0.429^***^	0.379^***^	0.413^***^	0.530^***^	—								
9. FPQ_MD	0.331^***^	0.374^***^	0.216^***^	0.660^***^	0.260^***^	0.354^***^	0.394^***^	0.445^***^	—							
10. MiniK	0.076^*^	0.080^*^	−0.005	−0.015	−0.018	0.051	0.006	0.093^**^	−0.029	—						
11. ACQ_EC	−0.181^***^	−0.197^***^	−0.082^*^	−0.147^***^	−0.164^***^	−0.269^***^	−0.149^***^	−0.211^***^	−0.207^***^	0.260^***^	—					
12. ACQ_TC	−0.201^***^	−0.204^***^	−0.121^***^	−0.197^***^	−0.202^***^	−0.257^***^	−0.185^***^	−0.202^***^	−0.289^***^	0.175^***^	0.413^***^	—				
13. ACQ_SC	−0.223^***^	−0.223^***^	−0.073^*^	−0.169^***^	−0.183^***^	−0.263^***^	−0.168^***^	−0.234^***^	−0.243^***^	0.112^***^	0.554^***^	0.584^***^	—			
14. MAS_SI	0.175^***^	0.191^***^	0.125^***^	0.218^***^	0.223^***^	0.287^***^	0.257^***^	0.178^***^	0.198^***^	−0.131^***^	−0.180^***^	−0.233^***^	−0.192^***^	—		
15. MAS_BI	0.225^***^	0.223^***^	0.107^**^	0.613^***^	0.215^***^	0.235^***^	0.428^***^	0.247^***^	0.495^***^	−0.068^*^	−0.138^***^	−0.231^***^	−0.173^***^	0.599^***^	—	
16. Knowledge	−0.043	−0.240^***^	0.003	−0.238^***^	−0.012	−0.100^**^	−0.214^***^	−0.290^***^	−0.209^***^	0.008	0.029	0.069^*^	0.049	−0.018	−0.109^***^	—
17. Experience	−0.132^***^	−0.219^***^	−0.114^***^	−0.181^***^	−0.098^**^	−0.073^*^	−0.197^***^	−0.249^***^	−0.153^***^	0.050	0.006	0.085^*^	0.063	0.045	−0.070^*^	0.279^***^

^*^p < 0.05, ^**^p < 0.01, ^***^p < 0.001. DS, Disgust sensitivity core; AR, animal remainder contamination; MFS, Medical Fear Survey; IDB, injection and blood draw; SO, sharp objects; ES, examinations and symptoms; BL, blood; MU, mutilation; FPQ MedDent, Fear of Medical/Dental Pain; Mini-K, Life history strategy variation; Perceived Control (ACQ) emotion (EC), threat (TC), and stress (SC); MAS, Medical Avoidance.

Taken together, our results are in line with our hypotheses. Former negative medical experience, lower levels of perceived control, higher levels of fear of medical pain, and a faster life history strategy were associated with a higher level of disgust sensitivity. Lower levels of health-related knowledge, fear of pain and disgust sensitivity were linked to a heightened fear of medical situations. Fear of pain, a perceived lack of control, and higher medical fears were associated with a higher probability of avoiding medical care.

## Discussion

It has been proposed that adaptive defense mechanisms facilitate recognition and appropriate responses, such as avoidance behaviors to potential environmental threats to reduce the probability of getting injured (1–3). However, the avoidance mechanisms may also be triggered by medical interventions and procedures, when avoidance is more harmful. Medical-related fears are associated with excessive distress toward stimuli commonly present in medical settings (33), are highly common (30), and have the most serious potential consequences (36) as they often result in the procrastination of seeking medical help (31). Therefore, the our study aimed to evaluate the impact of medical fears and disgust on medical avoidance concerning individual differences in perceived control, medical knowledge, former experiences, and life-history strategies. Our results showed that more knowledge of and experience with medical settings, and slower life strategies were associated with higher levels of perceived control and less intense emotional reactions. A better ability to control affective and stress reactions to negative experiences was linked to reduced disgust sensitivity and lower levels of fear of pain. These factors might mitigate the level of perceived threat, diminish fear and disgust reactions, and ultimately, might result in a decreased probability of avoiding medical screenings, examinations, and care. This is in line with the disease-avoidance model (16), Reinforcement Sensitivity Theory (20, 21), and fear-avoidance models (52) drawing a complex picture about the underlying mechanisms and motivations related to the avoidance of medical settings.

Our results underline the importance of examining the role of contextual factors, showing that life-history strategies developed in more uncontrollable and unpredictable environments, were associated with an elevated sense of control and disgust sensitivity. That is, less impulsive behavioral strategies and more predictable conditions during childhood are connected to increased behavioral control and more intense vigilance toward environmental cues of pathogens indicating stronger disease avoidance reactions. Similarly, medically relevant experiences and knowledge were associated with decreased disgust sensitivity and both fear of pain and medical fears, suggesting that direct and perhaps indirect encounters with environmental threats shape the frequency of avoidance reactions. These findings are in line with existing models of behavioral defense mechanisms (17, 19, 20) and support the notion, that avoidance behavior is triggered by individual evaluation processes based on the interpretation of former and actual circumstances. The current COVID-19 pandemic can be a good example of that: fears of illness and death were amplified, social distancing increased isolation (71) and these changes might intensified avoidant tendencies, which in turn reinforced the perceived threat of medical settings (e.g., higher chance to be infected if visiting the doctor). Further, we also found that perceived control of emotional reactions and threats was connected to medical avoidance through direct and indirect pathways, that is, better control was linked to a lesser likelihood of medical avoidance and reduced disgust sensitivity and fear of pain. It has been posited that a better ability to control affective and stress reactions to negative experiences alters pain processing (46) and therefore, mitigates the level of perceived threat, resulting in diminished fear and disgust reactions (47, 52). However, in medical settings perceived control might be decreased and its influence on avoidance less articulated due to the potential defenselessness of the individual (55).

Regarding disgust sensitivity, our findings suggest that different aspects of disgust may reflect a shared vulnerability for medical fears and avoidance. Accordingly, proneness to disgust may be characterized by harm avoidance, in general, and can be considered as a relevant trigger for medical avoidance in particular. This is in line with the disease-avoidance model that describes the functional role of disgust in the avoidance of contamination and protection against infections (16, 37). These results are also in agreement with former studies and show that disgust, as a response to potential health-related threats, affects avoidance behavior differently compared to fear (37, 38). Previous research suggests that people with BII phobia report greater disgust sensitivity than non-phobics, that exposure to stimuli related to medical and settings, and BII increases disgust more for phobic compared to non-phobic participants, and that disgust returns more rapidly after extinction than fear (72). As suggested by the disgust law of contagion of magical belief systems, things that were once in contact always retain that connection (73). Disgust intensifies the perception of the likelihood of negative or harmful consequences (74) elevating both the level of fear and the probability of avoidance in medical settings. Further, fear of pain and medical fears directly impact medical avoidance, which link was supported by several former studies as well (33, 75, 76). Hence, reducing medical avoidance might be a constant battle against the reoccurrence of beliefs and a strong urge of avoiding contamination and pain. A slower LHT might be useful in helping an individual to see the long-term payoff of a situation rather than the immediate danger.

Besides its significant contributions, the current study also has a few limitations. Longitudinal data would have been more informative (e.g., by measuring how avoidance behavior changes over time), and there might be other indicators that contributed to the probability of avoiding medical care. Further work is needed to explore and validate the different behavioral aspects of disgust and fear on medical avoidance and to analyse age-related effects in more detail. Further, external factors such as the form of medical care or severity of former injuries and diseases probably affect medical avoidance and might alter the connections but were not evaluated in the current study. Similarly, COVID-19-related worries and fears might affect medical avoidance but were not assessed in the current study. Although at the time of data collection, there were no measures imposed by governments to limit interpersonal contacts or medical visits, future studies should examine if the pandemic has any long-term effects on people's attitudes toward seeking medical help. Age may be also associated with the quality and severity of medical experiences, fear, and avoidance, but our study could not reveal this connection as the age range of our sample was not wide enough. Furthermore, the effects of the internal factors included in our study on actual avoidant behaviors should be clarified in practice, e.g., in real medical settings. In sum, internal contextual factors (reflected in experiences, knowledge, sense of control, disgust, and fear) affect individual medical avoidance tendencies. Thus, it is crucial to reflect on these factors in order to enhance the motivation of people to regularly attend medical screenings and seek medical help if needed. Elevated avoidance spoils adherence and favors the development of fear-related disorders (e.g., phobias) together with distortions in perceptual and decisional processes. Despite the health-related importance of pain and medical interventions, these biased cognitive-affective processes may prohibit the individual to invoke adequate help because of the activation of avoidance as part of the behavioral harm avoidance. Thus, an adaptive behavioral strategy might produce a maladaptive outcome and causing more harm to the individual than good.

## Data availability statement

The raw data supporting the conclusions of this article will be made available by the authors, without undue reservation.

## Ethics statement

The studies involving human participants were reviewed and approved by the Hungarian United Ethical Review Committee for Research in Psychology. The patients/participants provided their written informed consent to participate in this study.

## Author contributions

BB: conceptualization, methodology, writing–original draft and editing, and writing–review and editing. BK: investigation. CC: writing–original draft and editing and writing–review and editing. AZ: formal analysis, funding acquisition, methodology, writing–original draft and editing, and writing–review and editing. All authors contributed to the article and approved the submitted version.
